# Advances in allogeneic hematopoietic stem cell transplantation for Langerhans cell histiocytosis in children

**DOI:** 10.3389/fimmu.2025.1345855

**Published:** 2025-01-28

**Authors:** Guangqiang Meng, Saran Feng, Yan Wang

**Affiliations:** Department of Hematology, The First Affiliated Hospital of Shandong First Medical University & Shandong Provincial Qianfoshan Hospital, Jinan, Shandong, China

**Keywords:** Langerhans cell histiocytosis, allogeneic hematopoietic stem cell transplantation, myeloablative conditioning, reduced intensity conditioning, chimerism

## Abstract

Langerhans cell histiocytosis (LCH) is a disease caused by clonal expansion of CD1a+/CD207+ cells and is characterized by organ involvement and dysfunction. Treatment of LCH in children is risk-adapted, and multisystem LCH requires systemic therapy. Although systemic treatments such as chemotherapy and BRAF/MEK inhibitors have improved the cure rate of LCH, disease reactivation rates remain 30%, and eventually some patients progress to relapse-refractory LCH. Allogeneic hematopoietic stem cell transplantation (allo-HSCT) is a promising salvage treatment strategy for children with relapse-refractory LCH. However, many questions such as the efficacy and indications of allo-HSCT, as well as suitable conditioning regimen are still undetermined for children with LCH. This review aimed to provide an update on advances in allo-HSCT for LCH in children, including indications, stem cell sources, conditioning regimens, chimerism, transplant-related complications, outcomes, and treatment of relapse.

## Introduction

Langerhans cell histiocytosis (LCH) is a rare proliferative disease of the Langerhans cells (1–3). LCH has a wide spectrum of clinical presentations, ranging from solitary benign bony lesions to multi-organ involvement and dysfunction, which pose a high risk of death despite aggressive treatment. Depending on the number of organs or systems involved, LCH can be divided into single system LCH (SS-LCH) and multi-system LCH (MS-LCH). LCH with risk organs (RO-LCH) refers to infiltration of vital risk organs (RO) such as bone marrow, spleen, and liver. MS-LCH is divided into MS-RO+ and MS-RO- according to whether it involves the RO. The treatment of LCH in children varies depending on the severity of the disease (4–6). Poor prognostic factors include age < 2 years, RO involvement, relapse-refractory patients, and complications of hemophagocytic lymphohistiocytosis (HLH) or associated malignancies (7–10). Especially, LCH associated with another hematological neoplasm (LCH-AHN) were reported more frequently. Because LCH may associate in both children and adults to clonally related myeloid malignancies (11). High-risk LCH refers to the presence of the above risk factors. High-risk LCH have poor outcomes, and their 2-year survival rates are < 30% (12). Treatment for these high-risk LCH patients has not yet been established, but allogeneic hematopoietic stem cell transplantation (allo-HSCT) has curative potential for these patients.

Allo-HSCT for LCH treatment has a short history since 1987, with only a limited number of reported cases. The main reason for this is the low incidence of LCH. Approximately 5–6 children per million are diagnosed annually, and more than 50% of the patients are between 1 and 15 years of age, with a peak between 1 and 4 years (13). The reported incidence in adults is lower, approximately 1–2 individuals per million (14). The disease was previously considered non-malignant, and allo-HSCT is controversial. Recent studies have found that LCH is an inflammatory neoplasm of myeloid origin (15–17), and the reduction of transplant-related mortality after reduced intensity conditioning (RIC), more patients with LCH have been treated with allo-HSCT than before. Moreover, with an understanding of the pathogenesis of LCH, patients with BRAF-V600E and MAPK pathway (RAS-RAF-MEK-ERK) mutations accounted for a large proportion of LCH (18–20). Although inhibitors targeting these gene mutations are not curative, and they should be employed for an undetermined time in children, with currently unknown long-term consequences. Inhibitors have been used in clinical practice and have shown satisfactory therapeutic effects (18–20). As a result, the status of allo-HSCT in LCH has been challenged and the number of patients receiving allo-HSCT has decreased, especially in adults with LCH. However, for high-risk and relapse refractory LCH, allo-HSCT remains an effective salvage treatment for LCH.

## Indication of allogeneic hematopoietic stem cell transplant

Currently, there are two promising treatment strategies for patients with high-risk and relapse/refractory LCH. The first is a combination of 2-chlorodeoxyadenosine(2-CDA) and cytarabine(Ara-C) (21–23). Another promising approach is allo-HSCT because of its strong immunomodulatory effects and the ability to eradicate tumor cells. MS-RO+ and failure of conventional therapy have very poor outcomes (7, 8). For these patients, salvage approaches based on novel agents such as 2-CDA alone or in combination with Ara-C did not result in improved survival rates. Therefore, allo-HSCT has recently been used as an alternative salvage treatment. In LCH, organ dysfunction including that of the liver and bone marrow, and central nervous system involvement/neurodegeneration are associated with a higher mortality rate. Therefore, for repeated relapses of LCH, it is recommended that patients undergo allo-HSCT early to prevent irreversible organ damage and poor survival. In addition, allo-HSCT is also a promising treatment strategy for patients with LCH and hematologic malignancies, especially acute myeloid leukemia(AML) (24–28).

**Figure 1:**
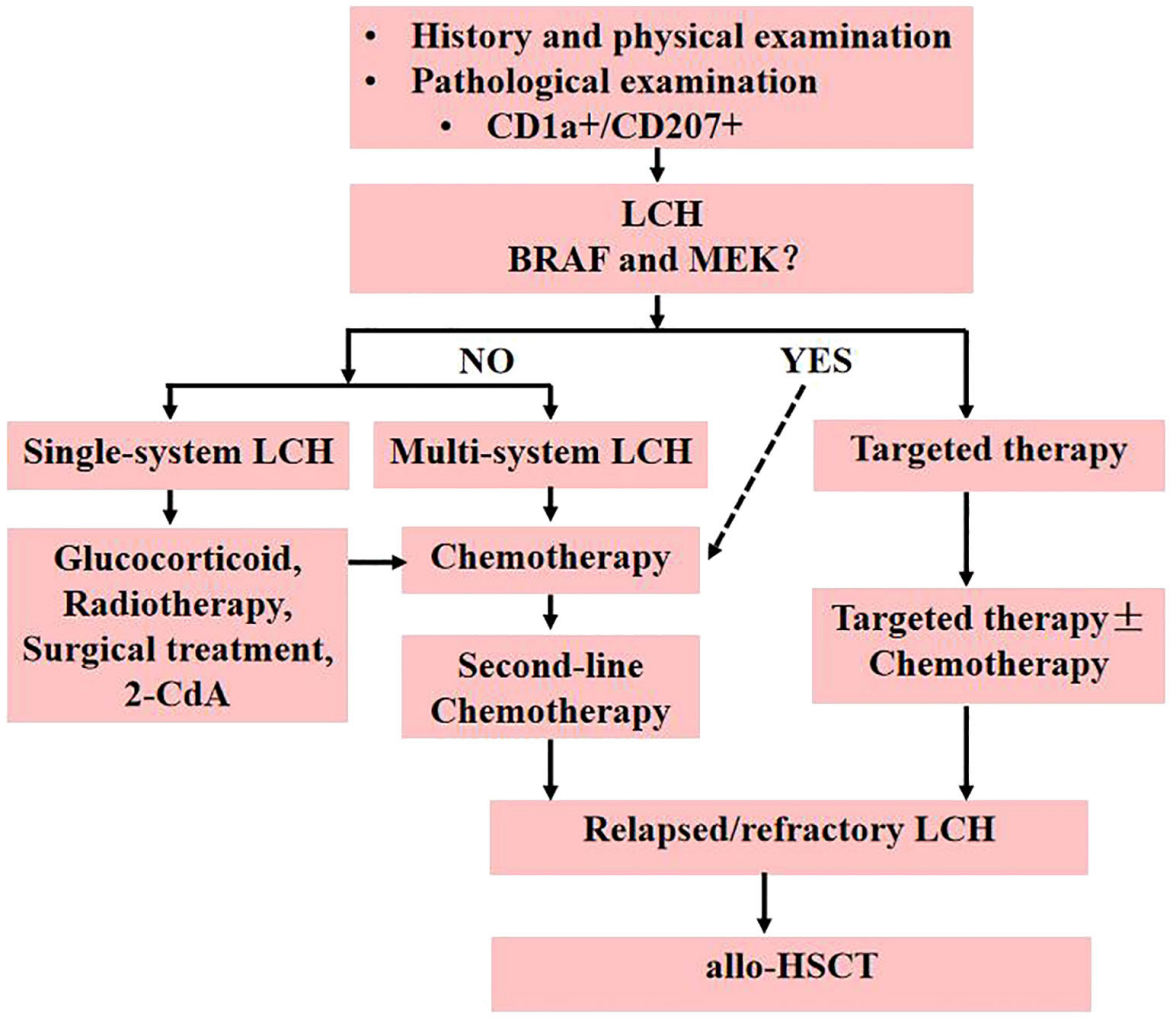
Treatment algorithm for children with LCH.

Up to 55% of patients with LCH have BRAF mutations, especially in children younger than 2 years (29–31). Therefore, molecular-targeted therapies are likely to provide more opportunities for LCH treatment (32). Vemurafenib (VMF), a BRAF inhibitor, has been used to treat refractory childhood LCH that was BRAFV600E mutation-positive. A total of 54 children participated in the clinical trial (33). At 8 weeks, 38 children achieved complete responses and 16 achieved partial responses. VMF seemed to be effective in children with refractory LCH BRAFV600E-positive. However, discontinuation of VMF in 30 children resulted in 24 cases of LCH reactivation. The median time to reactivation of LCH was 0.9 months, and the reactivation rates at 6 months and 12 months were 72% and 84%, respectively. Therefore, molecular targeted therapies cannot eradicate the BRAFV600E Clone. Molecularly targeted therapy diminishes the status of allo-HSCT; however, allo-HSCT can still be considered for children with gene-negative LCH and repeated relapses. Therefore, molecular targeted therapies can be used as a treatment method for bridging allo-HSCT in LCH patients, and those who fail inhibitor treatment should receive allo-HSCT as soon as possible. In conclusion, allo-HSCT is suitable for relapsed and refractory LCH in children who have failed chemotherapy and targeted therapy (**Figure 1**).

## Donor and stem cell source

Because the number of reported cases is limited, the best method and optimal time for transplantation are still not defined. Stem cell donors included matched family donors, matched unrelated donors, haploidentical donor, and unrelated cord blood donors. Due to the limited number of cases of children with LCH receiving HSCT and the lack of randomized controlled clinical studies, there is no clear and uniform conclusion on which donor is more beneficial to children. Compared with the stem cell source donor, the current researchers are more concerned about whether the stem cell source is bone marrow, peripheral blood or cord blood, because they are more likely to affect the efficacy and survival after transplantation. In earlier studies, most studies on transplantation for LCH have focused on bone marrow blood as the source of stem cells, whereas only a small proportion of studies have reported the use of peripheral and cord blood; However, in recent years, the number of applications of peripheral blood and cord blood stem cells has gradually increased. For example, of the 87 patients with LCH from the International Blood and Bone Marrow Transplantation Research Center (CIBMTR) and European Blood and Bone Marrow Transplant (EBMT), 20 underwent allo-HSCT before 2000 (34). There were 16 patients underwent bone marrow stem cell transplantation, 2 underwent peripheral blood stem cell transplantation, and 2 underwent cord blood stem cell transplantation. After the year 2000, 67 patients underwent allo-HSCT, 32 patients underwent bone marrow stem cell transplantation, 14 underwent peripheral blood stem cell transplantation, and 23 underwent cord blood stem cell transplantation.

At present, the application of peripheral blood and cord blood stem cells is also more favored, mainly because they have their own advantages. The infusion of peripheral blood stem cells containing a large number of CD34+ cells can result in more rapid hematological recovery (35). In addition, donor T lymphocytes in peripheral blood can have a stronger graft versus tumor (GVT) effect, which is important for patients with advanced-stage hematological malignancy (36). For infants, cord blood has many advantages such as an increased chance of suitable donors, rapid availability from banks, low risk of severe graft-versus-host disease (GVHD), and a low probability of transmitting infectious diseases. Most patients with LCH who require allo-HSCT are younger than 2 years, and cord blood can provide a large number of stem cells, leading to rapid engraftment and hematopoietic reconstitution (37–40). The number of patients eligible for some groups was small; therefore, it was difficult to comment on the efficacy and success of the different sources of hematopoietic stem cells.

## Conditioning

The conditioning regimens of allo-HSCT in patients with LCH reported in the current study were mainly myeloablative conditioning (MAC) and reduced intensity conditioning (RIC). The specific drugs for MAC and RIC are consistent with those for other hematological malignancies, and no special new drugs have been added. For example, the MAC regimens mainly contains: busulfan, cyclophosphamide or fludarabine, and some children also choose the total body irradiation (TBI) containing regimens; The RIC mainly contains melphalan and fludarabine containing regimens (34). The choice of conditioning regimens for allo-HSCT in patients with LCH has been an important issue in recent years, and the best choice remains uncertain.

Some studies have found that the advantage of MAC regimens is that it can eliminate LCH cells in the body as much as possible to reduce the recurrence of the disease. For example, Veys et al. ‘s study found that the 3-year disease recurrence rate was only 3% among 41 children who received MAC regimens from 2000 to 2013, while 28% among 25 children who received RIC regimens (34). And the 3-year Disease-free survival of the two regimens was 77% and 51%, respectively (34). However, MAC regimens are often associated with high treatment-related morbidity (TRM) (41). In a retrospective study, Veys et al. summarized 29 pediatric patients with RO-LCH who underwent myeloablative allo-HSCT (34). The overall survival rate was 48%, and TRM was exceedingly high at 45%. Preexisting disease-related organ (the liver, bone marrow, and lungs) dysfunction together with infection in the majority of patients seemed to be the main cause of high TRM. And Kesik et al. reviewed 44 children with refractory LCH who underwent allo-HSCT (42). Of the 18 children with fatal outcomes, 16 received myeloablative allo-HSCT, while only 2 patients who were treated with RIC died. Death was attributed to recurrence, treatment-related toxicity, and GVHD. Owing to the high TRM of MAC transplantation, the number of patients undergoing RIC transplantation for LCH has increased in recent years. Steiner et al. reported nine children with high-risk LCH treated with RIC allo-HSCT (41). The conditioning was well tolerated, and an encouraging result was obtained; seven patients without disease progression were alive at a median follow-up of 390 days after transplantation. Good outcomes after RIC allo-HSCT have also been confirmed in patients with LCH (43, 44). Therefore, in recent years, RIC’s regimens has been gradually recommended more and more. However, Kudo et al. also found that there was no difference in overall survival (OS) or failure-free survival between children with RIC and MAC (45). So, for children with LCH, the choice between MAC and RIC transplantation requires further clinical research data to be confirmed. In clinical studies on HSCT in children with LCH, the conditioning regimens and outcomes are shown in **Table 1**.

**Table 1:** Cohort studies reporting allo-HSCT results in the treatment of LCH in children.

Number of Patients	Median Age	Conditioning Regimen	Incidence of aGVHD	Incidence of cGVHD	TRM	Relapse	OS Rate	Reference
20 (between 1990 and 1999)	2 years	MAC 18 RIC 2	Grade II-IV 45%	10%	55%	0	25%	(34)
41 (between 2000 and 2013)	2 years	MAC 41 RIC 26	Grade II-IV MAC 31%,RIC 9% Grade III-IV MAC 18%,RIC 9%	MAC 27% RIC 21%	MAC19.5%RIC19.2%	MAC8% RIC28%	MAC77% RIC71%	(34)
4	1.75 years, 11 months, 12.83 years, 7 months	BU/L-pam/CY, VP-16/CY/TBI, VP-16/CY/ATG, CY/TBI,	No information	No information	50%	0	50%	(46)
5	0.54 years	VP-16/BU, VP-16/CY/BU, BU/CY, BU/CY, VP-16/TBI	60%	20%	40%	20%	40%	(47)
13	15 years	MAC 8 RIC 5	23.1%	15.4%	15.4%	15.4%	73.3%	(48)
9	9.9 months	RIC	11.1%	11.1%	11.1%	0	77.8%	(41)
30	1 years	MAC 11 RIC 19	MAC 36.4% RIC 36.8%	MAC 27.3% RIC 5.3%	MAC 36.4% RIC 36.8%	MAC 18.2% RIC 5.3%	MAC 63.6% RIC 56.8%	(45)

Allo-HSCT, allogeneic hematopoietic stem cell transplantation; LCH, Langerhans cell histiocytosis; aGVHD, acute graft-versus-host disease; cGVHD, chronic graft-versus-host disease; TRM, treatment-related morbidity; OS, overall surviva; MAC, myeloablative conditioning; RIC, reduced intensity conditioning; BU, busulfan; CY, cyclophosphamide; VP-16, etoposide; TBI, total body irradiation. ATG, anti-thymocyte globulin.

## Chimerism

Generally, the median time reported for successful neutrophil implantation after RIC allo-HSCT in patients with LCH is 14 days (12–49 days) (34). Successful red blood cell and platelet implantation typically occurs on 38 and 37 days after transplantation, respectively (34). Veys et al. found that hematopoietic recovery rates after MAC transplantation were not significantly different from those after RIC transplantation (34). However, recovery rates were slow for both MAC and RIC transplantations, with most patients undergoing implantation 2 months after transplantation. The disadvantage of RIC allo-HSCT is the increased risk of non-engraftment and graft rejection. In contrast to allo-HSCT after MAC conditioning, RIC allo-HSCT usually leads to mixed chimerism, which is defined as the coexistence of hematopoietic cells from the host and donor. However, that neither non-engraftment nor mixed chimerism leads to the deterioration of LCH and a life-threatening state, and does not necessarily implicate an exacerbation of LCH. Kinugawa et al. and Akkari et al. reported that two patients failed engraftment but achieved complete autologous recovery of hematopoiesis without disease activity (46, 47). Both patients were alive and disease-free at 3 and 12 years after transplantation at the time of the last follow-up. Steiner et al. reported five of nine patients had full donor chimerism on day +28 after RIC allo-HSCT, which persisted until the last follow-up (69–390 days after allo-HSCT) (41). Two patients exhibited mixed chimerism until the last follow-up. Steiner et al. also reported that one patient achieved complete remission after RIC allo-HSCT despite mixed chimerism after transplantation, in which only a T-cell subset proved to be of donor origin (41). There are two reasons for this phenomenon. Patients with LCH in a state of engraftment and mixed chimerism are in sustained remission after transplantation. On one hand, a highly immunosuppressive conditioning regimen together with GVHD prophylaxis may decisively contribute to the stabilization of the disease. In contrast, donor T-cells play an important role. The presence of donor T-cells is sufficient to restore normal immunoregulation and prevent hypercytokinemia and macrophage activation, which play important roles in the pathogenesis of LCH (36). Moreover, myeloablation is not critical for the treatment of LCH, although data on this disease are limited.

## Transplant-related complication

GVHD remains an important complication of allo-HSCT in patients with LCH. Prophylaxis drugs for GVHD and graft rejection consisted of ciclosporin A (CSA), mycophenolate mofetil (MMF), prednisone, and methotrexate (MTX) (34, 41). Acute GVHD was more common than chronic GVHD in patients with LCH. Veys et al. reported 18 patients who received MAC regimens, with period prior to 2000 (34). Grades II–III acute GVHD occurred in eight patients (two grade II and six grade III), and chronic GVHD occurred in two patients. One of the two patients who received the RIC regimen developed grade II acute GVHD. Akkari et al. reported that three out of five allo-HSCT recipients developed evidence of grade I or II acute GVHD, but transient limited chronic GVHD occurred in only one patient (47). Kudo et al. reported three of ten patients who underwent unrelated cord blood transplantation developed acute GVHD (grades I, III, and IV) (48). One of them and another patient developed chronic GVHD. In patients with LCH, acute GVHD often involves the gastrointestinal mucosa, liver, and skin. Acute GVHD and the subsequent limited chronic GVHD are often responsive to immunosuppressive therapy. Veys et al. retrospectively analyzed patients who underwent allo-HSCT between 2000 and 2013 (34). The incidence of grade II–IV acute GVHD was marginally higher after MAC regimens than after RIC regimens. However, there were no differences in the incidences of grades III–IV acute and chronic GVHD. Other transplant-related complications include infection, mucositis, liver damage, interstitial pneumonia, organ failure, and hypertension (34, 47, 48).

The incidence of GVHD after LCH transplantation in children is slightly different from that after transplantation for other childhood diseases. Veys et al. ‘s study found that the incidence of grade II-IV acute GVHD after LCH transplantation in 41 children after 2000 was 31%, while that of chronic GVHD was 27% (34). In a transplant study of 618 children with benign and malignant disease, Cumulative incidence of acute GvHD (I-IV) was 31.5% and chronic GvHD was12.8% (49). Thus, the incidence of acute GVHD in children after LCH transplantation is roughly the same as that of other childhood diseases, while the incidence of chronic GVHD is slightly higher. However, the number of cases of LCH transplantation studies in children is still small, and further confirmation is still needed in large sample studies. In terms of TRM of both studies, the incidence of LCH in children within 3 years after transplantation was 15%, compared to 31.2% in the median follow up of 115.1 months after transplantation for other childhood diseases (34, 49). Because of the different follow-up times, it is also difficult to compare the incidence of TRM after transplantation between LCH and other diseases.

## Survival and outcome

Chemotherapy failure occurs in approximately 20% of patients with multisystem LCH, with an overall survival of only 20% (7, 50). Allo-HSCT has recently been used in these patients as a salvage treatment to improve prognosis. Kudo et al. reported that 15 children younger than 15 years with refractory LCH underwent allo-HSCT (48). Eleven of the fifteen patients survived without evidence of disease, with a 10-year OS rate of 73.3%. The 10-year OS rate of nine patients with RO at diagnosis was 55.6%, and six patients without RO survived without evidence of disease. These results indicated that allo-HSCT is a promising salvage approach for children with refractory LCH. Three patients with LCH reported by Coope et al. treated with RIC allo-HSCT remained alive and in remission at a median of 5.1 years after allo-HSCT (43). Allo-HSCT can improve the prognosis of relapse and refractory LCH and prolong patient survival.

In addition, studies have found that the disease status of LCH during HSCT can affect a patient’s OS. Kudo et al. retrospectively analyzed 30 children with refractory LCH (45). The 5-year OS of children with no active disease and active disease regression was 100% after HSCT, significantly better than the 54.5% of children with active disease and active disease progression. This study suggests that LCH disease status at HSCT can affect OS. It is important for patients to achieve better remission with active and effective treatments before undergoing HSCT.

## Treatment for relapse after allo-HSCT

Compared with other treatments, the rates of relapse or progression were lower in patients with LCH after allo-HSCT. The treatment options for LCH recurrence after allo-HSCT include secondary transplantation and combined chemotherapy. Six patients reported by Veys et al. underwent a second transplantation due to non-engraftment (n=4) or recurrent disease (n=2) (34). Of the patients re-transplanted for non-engraftment, one received MAC and three received RIC regimens for their first transplantation. Two of the three patients who received the RIC regimen for the first transplantation were alive 2 and 9 years after their first transplantation. Of the two patients who received a second transplant for recurrent disease, one was alive 6 years after the second transplantation and the other died 18 months after the second transplant. Therefore, in patients with graft failure and recurrence after allo-HSCT, a second transplant can result in long-term survival. Veys et al. reported that four of six patients who relapsed after RIC allo-HSCT achieved further remission after receiving chemotherapy as a salvage treatment (30). Patients who are BRAF-positive and do not use targeted drugs can also try this targeted therapy.

## Conclusion and future prospectives

In summary, allo-HSCT is an important curative method for the relapsed/refractory LCH in children, especially for the failure of chemotherapy and targeted therapy. Due to the fact that LCH is a rare disease, and the research data of allo-HSCT are still few, there are still many problems that are still unclear and worth exploring, such as the indication and timing of allo-HSCT, the selection of preconditioning intensity and the treatment for relapse after allo-HSCT. In the future, we still need large, prospective, multineutral clinical studies to explore the unknown questions of allo-HSCT for LCH in children.
